# Multi-environment Genomic Prediction of Plant Traits Using Deep Learners With Dense Architecture

**DOI:** 10.1534/g3.118.200740

**Published:** 2018-09-28

**Authors:** Abelardo Montesinos-López, Osval A. Montesinos-López, Daniel Gianola, José Crossa, Carlos M. Hernández-Suárez

**Affiliations:** * Departamento de Matemáticas, Centro Universitario de Ciencias Exactas e Ingenierías (CUCEI), Universidad de Guadalajara, 44430, Guadalajara, Jalisco, México; † Facultad de Telemática, Universidad de Colima, 28040, Colima, México; ‡ Departments of Animal Sciences, Dairy Science, and Biostatistics and Medical Informatics, University of Wisconsin-Madison, 53706, Madison, Wisconsin; § International Maize and Wheat Improvement Center (CIMMYT), Apdo. Postal 6-641, 06600, Ciudad de México, México; ** Facultad de Ciencias, Universidad de Colima, 28040, Colima, Colima, México

**Keywords:** GBLUP, deep learning, neural network, genomic prediction, prediction accuracy, GenPred, Shared Data Resources

## Abstract

Genomic selection is revolutionizing plant breeding and therefore methods that improve prediction accuracy are useful. For this reason, active research is being conducted to build and test methods from other areas and adapt them to the context of genomic selection. In this paper we explore the novel deep learning (DL) methodology in the context of genomic selection. We compared DL methods with densely connected network architecture to one of the most often used genome-enabled prediction models: Genomic Best Linear Unbiased Prediction (GBLUP). We used nine published real genomic data sets to compare a fraction of all possible deep learning models to obtain a “meta picture” of the performance of DL methods with densely connected network architecture. In general, the best predictions were obtained with the GBLUP model when genotype×environment interaction (G×E) was taken into account (8 out of 9 data sets); when the interactions were ignored, the DL method was better than the GBLUP in terms of prediction accuracy in 6 out of the 9 data sets. For this reason, we believe that DL should be added to the data science toolkit of scientists working on animal and plant breeding. This study corroborates the view that there are no universally best prediction machines.

It is important to use new technologies to increase food production, given that the world population will reach 10.4 billion by 2067, with 81% residing in Africa or Asia. Due to the increase in population, there will be a decrease of 0.15 ha per person in the arable land available for food production. Further, temperature is expected to increase in tropical and temperate zones, especially in the Northern Hemisphere, which will push growing seasons and farming areas away from arid areas into more northern latitudes (Britt *et al.*, 2018). Under these scenarios, increasing world food production is a challenge. Genomic selection is a promising development in agriculture that aims to improve production by exploiting molecular genetic markers to design novel breeding programs and develop marker-based methods for genetic evaluation of plants and animals (Jonas and de Koning 2015; Hickey *et al.*, 2017).

Genomic selection (GS) is a type of marker-assisted selection that uses dense molecular markers from the entire genome simultaneously in a linear regression model (Meuwissen *et al.*, 2001). A predictive model using individuals with known genotypic and phenotypic information is then constructed. With this model, genomic estimated breeding values (GEBVs) for the desired trait are calculated and used to rank individuals with unknown phenotypes for subsequent selection. The accuracy of the predictions is evaluated using some form of cross-validation. Originally proposed in animal breeding, this method has revolutionized and transformed breeding programs worldwide, and is being implemented in most developed nations. The fast growing popularity of GS can be attributed to a continuous reduction in the cost of obtaining large numbers of DNA markers of plant or animal genomes, and to the empirical evidence that this approach indeed improves genetic gains per unit of time, facilitating the rapid selection of superior genotypes and accelerating the breeding cycles (Weller *et al.*, 2017).

For these reasons, genomic selection is being implemented by commercial companies and national breeding programs of maize and wheat (Crossa *et al.*, 2017), cassava (Wolfe *et al.*, 2017), oil palm (Kwong *et al.*, 2017), and macadamia (O’Connor *et al.*, 2018), among others. The goal of most breeding programs is to predict the genetic merit of unphenotyped individuals and thus enable targeted combinations of desired alleles to improve the performance of the next generation(s). However, to effectively implement GS in crop breeding also requires prediction models that can improve prediction accuracy in large-scale data sets and are robust across trait-environment combinations. Prediction models often perform poorly for some trait-environment combinations, so the search for better genomic prediction models is an active area of research.

Machine learning (ML) is a field of computer science that uses statistical techniques to give computer systems the ability to “learn” (*i.e.*, progressively improve performance on a specific task) from data, without being explicitly programmed to do this (Samuel 1959). ML is closely related to (and often overlaps with) computational statistics, which also focuses on making predictions through the use of computers. In general, ML explores algorithms that can learn from current data and make predictions on new data, by building a model from sample inputs (Samuel 1959). The fields of statistics and ML have some goals in common and will continue to come closer together in the future. Although applications of ML in genomic selection (González-Camacho *et al.*, 2012) exist, application of DL methods in genomic prediction is lacking.

This paper evaluates prediction accuracy in the context of genomic selection of Deep Learning (DL) methods with a densely connected network architecture, which is a type of ML algorithm that uses an artificial neural network with multiple layers linked nonlinearly. The “deep” in DL refers to the number of layers through which the data are transformed. The layers in these methods consist of multiple stages of nonlinear data transformations, where features of the data are represented by successively higher and more abstract layers. The goal of a DL method is either to predict or to classify a response variable using inputs. Traditional linear regression models are not considered deep because they do not apply multiple layers of non-linear transformations to the data. The prediction performance of DL methods has proved to be similar or better than that of traditional methods in many areas like health care, image processing, natural language processing, speech recognition, military target recognition, marketing, investment portfolio management, financial fraud detection, stock market forecasting, optical character recognition and traffic sign classification (Deng and Yu 2013). Also, companies such as Microsoft, Google, IBM, Yahoo, Twitter, Baidu, Paypal and Facebook are exploiting DL methods to understand consumers (Deng and Yu 2013).

There have been successful applications of DL in the biological sciences. For example, Menden *et al.* (2013) applied a DL method to predict the viability of a cancer cell line exposed to a drug. Alipanahi *et al.* (2015) used DL with a convolutional network architecture to predict specificities of DNA- and RNA-binding proteins. Tavanaei *et al.* (2017) used a DL method for predicting tumor suppressor genes and oncogenes. DL methods have also made accurate predictions of single-cell DNA methylation states (Angermueller *et al.*, 2017). In the area of genomic selection, we found two reports only: (a) McDowell and Grant (2016) found that DL methods performed similarly to several Bayesian and linear regression techniques that are commonly employed for phenotype prediction and genomic selection in plant breeding; (b) Ma *et al.* (2017) also used a DL method with a convolutional neural network architecture to predict phenotypes from genotypes in wheat and found that the DL method outperformed the GBLUP method.

In this study we examine a DL method with a densely connected network architecture in the context of GS in plants to have a better idea of its prediction performance. We compare the DL method with GBLUP, the most widely used method. Our study involved 9 multi-environment real data sets used in genomic selection of wheat and maize breeding programs. The data sets comprise a large number of wheat and maize lines with several traits that were measured in several environments.

## Materials and Methods

### Model implementation

#### Multiple-environment Genomic best linear unbiased predictor (GBLUP) model:

Since genotype×environment interaction is of paramount importance in plant breeding, the following univariate linear mixed model is often used for each trait:

$$y_{ij}=E_{i}+g_{j}+gE_{ij}+e_{ij}$$ (1)

where $y_{ij}$ represents the response of the jth line in the ith environment (i=1,2,...,I, j=1,2,...,J). $E_{i}\;$represents the fixed effect of the ith environment, $g_{j}\;$ represents the random genomic effect of the jth line, with $g={\left(g_{1},...,g_{J}\right)}^{T}∼N\left(0,\sigma_{1}^{2}\;G_{g}\right),$ $\sigma_{1}^{2}$ is a genomic variance and $G_{g}$ is of order J×J, represents the genomic relationship matrix (GRM) and is calculated (VanRaden 2008) as $G_{g}=\frac{WW^{T}}{p}$, where p denotes the number of markers and W is the matrix of markers of order J×p. The $G_{g}$ matrix is constructed using the observed similarity at the genomic level between lines, rather than the expected similarity based on pedigree. Further, $gE_{ij}$ is the random interaction term between the genomic effect of the jth line and the ith environment; let $\mathrm{gE}={\left(gE_{11},...,gE_{IJ}\right)}^{T}∼N\left(0,\sigma_{2}^{2}\;I_{I}⊗G\right)$, where $\sigma_{2}^{2}$ is an interaction variance, and $e_{ij}$ is a random residual associated with the jth line in the ith environment distributed as $N\left(0,\sigma^{2}\right),$ where $\sigma^{2}$ is the residual variance.

#### Deep learning model:

Popular neural network architectures are: (a) densely connected networks, (b) convolutional networks, and (c) recurrent networks. Details on each type of network, its assumptions and input characteristics can be found in Gulli and Sujit (2017), Chollet and Allaire (2017) and Angermueller *et al.* (2016). In this study we implemented type (a), which is a typical feedforward neural network also known as multilayer perceptron, which does not assume a specific structure in the input features (Goodfellow *et al.*, 2016). In general, the basic structure of a densely connected network consists of an input layer, an output layer and multiple hidden layers between the input and output layers. Neurons (units) are connected in the network; the strength of the connection between neurons is called weight. The weight values of the connections between the layers are how neural networks encode the learned information extracted from the raw training data. The input layer neurons correspond to the number of features (called independent variables by the statistics community) you wish to feed into the neural network. The hidden layer neurons are generally used to perform non-linear transformation of the original input attributes (Lewis 2016). The number of output neurons corresponds to the number of response variables (traits in plant breeding) you wish to predict or classify and they receive as input the output of hidden neurons and produce as output the prediction values of interest (Goodfellow *et al.*, 2016).

The layer is the core building block of a neural network; it is a data-processing step that we can think of as a filter for data, since the data that go in are transformed and come out in a more useful form. Specific layers extract representations out of the data, which are fed into representations that are more meaningful for the problem at hand. Most DL methods consist of joining together simple layers that will implement a form of progressive data distillation (Chollet and Allaire 2017).

In training neural networks, one epoch means one pass (forward and backward) of the full training set through the neural network. Since one epoch is too big to feed into the computer at one time, we divide it into several smaller batches. A batch consists of a number of training samples in one forward/backward pass. The larger the batch size, the more memory is needed to run the model. For example, suppose you had a batch size of 500, with 1000 training samples. It will take only two iterations to complete one epoch. An iteration is the number of batches needed to complete one epoch. We used more than one epoch because too few epochs lead to underfitting of DL models. Therefore, as the number of epochs increases, the weights are changed in the neural network and the DL model goes from underfitting to optimal fitting or to overfitting. Unfortunately, the right number of epochs is data dependent.

Also, due to the sensitivity of DL models to overfitting, constraints are put on the complexity of a neural network by forcing its weights to take on only small values, which makes the distribution of weight values more regular. This is called weight regularization, and it is done by adding to the loss function of the network a cost (penalty) associated with having large weights. There are many types of regularization but in this paper we implemented dropout regularization, which consists of temporarily removing a random subset (%) of neurons with their connections during training. This means that their contribution to the activation of downstream neurons is temporarily removed on the forward pass and any weight updates are not applied to the neurons on the backward pass. In other words, dropout consists of randomly dropping out (setting to zero) a number of output features of the layer during training. Unfortunately, choosing the optimal values for each of these hyperparameters is challenging; the process of choosing these values is art and science.

##### Model selection in DL.

Hyperparameters govern many aspects of the behavior of DL models, such as their ability to learn features from data, the models’ exhibited degree of generalizability in performance when presented with new data, as well as the time and memory cost of training the model, since different hyperparameters often result in models with significantly different performance. This means that tuning hyperparameter values is a critical aspect of the model training process and a key element for the quality of the resulting prediction accuracies. However, in DL models, making a good choice of the number of layers, number of units (neurons), number of epochs, type of regularization penalty, type of activation function, among others is challenging.

Manual tuning of DL models is of course possible, but relies heavily on the user’s expertise and understanding of the underlying problem. Additionally, due to factors such as time-consuming model evaluations, non-linear hyperparameter interactions in the case of large models, and tens or even hundreds of hyperparameters, manual tuning may not be feasible. For this reason, the four most common approaches for hyperparameter tuning reported in the literature are: (a) grid search, (b) random search, (c) Latin hypercube sampling, and (d) optimization (Koch *et al.*, 2017). In the grid search method, each hyperparameter of interest is discretized into a desired set of values to be studied, and models are trained and assessed for all combinations of the values across all hyperparameters (that is, a “grid”). Although fairly simple and straightforward to carry out, a grid search is quite costly because the expense grows exponentially with the number of hyperparameters and the number of discrete levels of each.

A random search differs from a grid search in that we no longer provide a discrete set of values to explore for each hyperparameter; rather, we provide a statistical distribution for each hyperparameter from which values may be randomly sampled. This allows a much greater chance of finding effective values for each hyperparameter. While Latin hypercube sampling is similar to the previous method, it is a more structured approach because it uses a random Latin hypercube sample (LHS) (McKay 1992), an experimental design in which samples are exactly uniform across each hyperparameter but random in combinations. These so-called low-discrepancy point sets attempt to ensure that points are approximately equidistant from one another in order to fill the space efficiently. This sampling allows for coverage across the entire range of each hyperparameter and is more likely to find good values of each hyperparameter.

The previous two methods for hyperparameter tuning perform individual experiments by building models with various hyperparameter values and recording the model performance for each. Because each experiment is performed in isolation, this process is parallelized, but is unable to use the information from one experiment to improve the next experiment. Optimization methods, on the other hand, consist of sequential model-based optimization that allows using the results of previous experiments to improve the sampling method of the next experiment. These methods are designed to make intelligent use of fewer evaluations and thus save on the overall computation time (Koch *et al.*, 2017). Optimization algorithms that have been used in machine learning generally for hyperparameter tuning include Broyden-Fletcher-Goldfarb-Shanno (BFGS) (Konen *et al.*, 2011), covariance matrix adaptation evolution strategy (CMA-ES) (Konen *et al.*, 2011), particle swarm (PS) (Renukadevi and Thangaraj 2014), tabu search (TS), genetic algorithms (GA) (Lorena and de Carvalho 2008), and more recently, surrogate-based Bayesian optimization (Dewancker *et al.*, 2016). Also, recently the use of the surface response methodology has been explored for tuning hyperparameters in random forest models (Lujan-Moreno *et al.*, 2018). However, the implementation of these optimization methods is not straightforward because it requires expensive computation; also, software development is required for implementing these algorithms automatically. There have been advances in this direction for some machine learning algorithms in the statistical analysis system (SAS) software (Koch *et al.*, 2017). An additional challenge is the unpredictable computation expense of training and validating predictive models using different hyperparameter values. Finally, although it is challenging, the tuning process often leads to hyperparameter settings that are better than the default values, since it provides a heuristic validation of these settings, giving greater assurance that a model configuration that has higher accuracy has not been overlooked.

##### Real data sets.

Three maize and six wheat data sets were analyzed.

#### Maize data sets 1-3:

These three data sets are made up of a total of 309 maize lines which were used by Crossa *et al.* (2013) and Montesinos-López *et al.* (2016, 2017). Traits evaluated were grain yield (GY; data set 1), anthesis-silking interval (ASI; data set 2), and plant height (PH; data set 3); each of these traits was measured in three environments (Env1, Env2, and Env3). Phenotypes of each trait were pre-analyzed and adjusted for the experimental field design. The number of single nucleotide polymorphisms (SNP), after filtering for missing values and minor allele frequency, was 158,281.

#### Wheat data sets 4-6:

These three data sets were used by López-Cruz *et al.* (2015) and Cuevas *et al.* (2016). The phenotypes in the three data sets are grain yield (GY, tons/hectare) adjusted for the experimental design. The data sets came from CIMMYT and were obtained from its wheat breeding station at Cd. Obregon, Sonora, Mexico. The environments were three irrigation regimes (moderate drought stress, optimal irrigation, and drought stress), two planting systems (bed and flat planting), and two different planting dates (normal and late). Wheat data set 4 had 693 wheat lines evaluated in four environments; wheat data set 5 included 670 wheat lines evaluated in four environments, and wheat data set 6 had 807 lines evaluated in five environments. Genotypes were derived using genotype by sequencing (GBS) technology; in all the analyses we used 15,744 GBS markers that resulted after quality control.

#### Wheat data sets 7-8:

These two wheat data sets came from a total of 250 wheat lines that were extracted from a large set of 39 yield trials grown during the 2013-2014 crop season in Ciudad Obregon, Sonora, Mexico (Rutkoski *et al.*, 2016). The traits measured were: (1) plant height (PH) recorded in centimeters (data set 7), and (2) days to heading (DTHD) recorded as the number of days from germination until 50% of spikes had emerged in each plot (data set 8), in the first replicate of each trial. Phenotypes were adjusted by experimental design as well. The genomic information was obtained by GBS and we used a total of 12,083 markers that remained after quality control.

#### Wheat Iranian data set 9:

This data set was used in Crossa *et al.* (2016), where full details are presented. It consists of 2374 wheat lines evaluated in a drought environment (D) and a heat environment (H) at the CIMMYT experiment station near Ciudad Obregón, Sonora, Mexico (27 ° 20 ’N, 109 ° 54 ’W, 38 meters above sea level), during the 2010-2011 cycle. The measured trait was days to maturity (DTM). The number of markers used was 39,758 that remained after the quality control process from a total of 40,000 markers.

##### Method implementation.

The GBLUP method was implemented with the BGLR package (de los Campos and Pérez-Rodríguez 2014) in the R statistical software (R Core Team 2018). DL methods were fitted with the Keras package (Gulli and Sujit 2017; Chollet and Allaire 2017) with a densely connected network architecture also in the R statistical software. In both GBLUP and DL, we used two different sets of covariates: the first set was composed of information on environments and genomes (that takes into account genomic information), while the second set of covariates included genotype×environment interaction (G×E) information as well. It is important to point out that marker information was not included directly as covariates in both models (DL and GBLUP) since information on markers was included in the design matrix of genotypes and G×E through Cholesky decomposition of the genomic relationship matrix (GRM) that was calculated with the marker information as mentioned above with the VanRaden (2008) method. The GBLUP and DL models were compared with and without the G×E term. Since the DL method requires values of some tuning parameters, we first ran several DL scenarios by choosing as tuning parameters some values recommended in the DL literature. Based on such runs, we implemented the grid search method with a full factorial design with the following three factors: (a) number of units (U), (b) number of epochs (E), and (c) number of layers (L). For U we used 50, 60, 70, 80, 90 and 100; for E we used 20, 40, 60, 80 and 100; and for L we used 1, 2 and 3. Thus 6×5×3 = 90 experiments were run for each data set with a densely connected DL method. It is important to point out that the 90 DL experiments used dropout regularization, which is one of the most effective and commonly used regularization techniques in neural networks, developed by Srivastava *et al.* (2014) at the University of Toronto. In our case, the dropout rate was fixed at 0.3 (30%); this meant that the percentage of features that were set to zero was 30% in each layer; this value was selected following the suggestions of Gulli and Sujit (2017), Chollet and Allaire (2017) and Srivastava *et al.* (2014). Also, concerning the activation function we implemented in the deep layers and output layer the Rectified linear unit (Relu).

#### Cross-validation:

Prediction accuracy of both DL and GBLUP was evaluated with random cross-validation (CV): the whole data set was divided into a training (TRN) and a testing (TST) set. This cross-validation is the same as the so-called replicated TRN-TST in the publication of Daetwyler *et al.* (2012) since some individuals can never be part of the training set. The percentages of the whole data set assigned to the TRN and TST sets were 65% and 35%, respectively. Our random CV used sampling with replacement, which means that one observation can appear in more than one partition. The design we implemented mimics a prediction problem faced by breeders in incomplete field trials where lines are evaluated in some, but not all, target environments. More explicitly, TRN-TST partitions were obtained as follows: since the total number of records per trait available for the data set with multi-environments is N=J×I, to select lines in the TST data set, we fixed the percentage of data to be used for TST (PTesting = 35%). Then we chose 0.35×N (lines) at random, and subsequently, one environment per line was randomly picked from I environments. The resulting cells (ij) were assigned to the TST data set, while cells not selected through this algorithm were allocated to the TRN data set. Lines were sampled without replacement if J≥0.35×N, and with replacement otherwise (Lopez-Cruz *et al.*, 2015).

The cross-validation we just described is called the outer CV and was applied for both models. However, in the DL model, an inner CV strategy was also applied for tuning the hyperparameters using the grid of hyperparameter values defined above (90 experiments). The inner CV strategy consisted of splitting each training set of the outer CV, where 20% of data were assigned to testing-inner and 80% to training-inner.The training-inner data set was used to train the DL model using the grid of hyperparameter values. This inner CV strategy was facilitated by using the internal capabilities of Keras and the validation_split argument on the fit() function. The predictive power is assessed in the second part of the data set (testing-inner). With this, a set of best-fitting hyperparameters (the best combination of units, epochs and layers) from the inner CV loop is obtained. Finally, this set of hyperparameters was used to predict the performance of the independent testing data set (testing-outer). For each data set, 10 random outer CV partitions were implemented, and with the observed and predicted values of each testing-outer data sets, we calculated the average Pearson’s correlation as a measure of prediction accuracy. It is important to point out that the outer cross-validation we implemented did not allow forward prediction because our TRN and TST sets were not separated across generational lines (Daetwyler *et al.*, 2012). The accuracy reported in terms of Pearson’s correlation was divided by the square root of the heritability of each trait-environment combination since heretabilities change in each trait-environment combination.

##### Data availability and software.

The phenotypic and genotypic data used in this study can be found in several articles (see the description of the data above). The readers can download the DataSets_DK.rar used in this study from the following link hdl:11529/10548082. Furthermore, R codes for fitting the DL methods used in this study are given in the Appendix.

## Results

The results are given in 10 sections, one for each real data set plus one where all data sets are compared. In the first 9 sections, we provide a figure with the predictions disaggregated by environment obtained with the DL model and those of the GBLUP model. For the DL model, the predictions reported correspond to the best combination (in terms of epochs, layers and units) obtained from the grid search. Finally, in Figure 10 we provide a meta-picture of the prediction performance of the 9 data sets, where the prediction performance of the best DL model is compared to that of the GBLUP model across environments in each data set.

### Maize data set 1-trait GY

**Figure 1:**
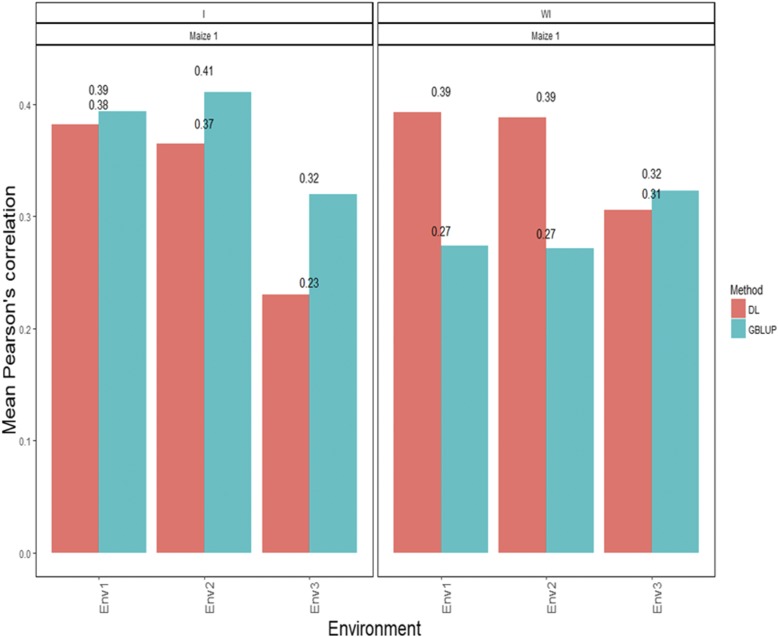
*Maize data set 1-Trait GY*. Mean Pearson’s correlation for each environment. The first vertical sub-panel corresponds to the model with genotype×environment interaction (Maize data set 1 I), and the second vertical sub-panel corresponds to the same model but without genotype×environment interaction (Maize data set 1 WI).

Figure 1 shows that the average Pearson’s correlation (APC) prediction accuracies under the GBLUP method desegregated by environment when the G×E term interaction was taken into account were: 0.394 for environment 1, 0.411 for environment 2 and 0.319 for environment 3. The predictions (Figure 1) under the DL method were: 0.382 for environment 1, 0.365 for environment 2 and 0.230 for environment 3. When the covariates corresponding to the G×E interaction term were ignored in both methods, the APCs were 0.274 for environment 1, 0.272 for environment 2 and 0.323 for environment 3 under the GBLUP method. On the other hand, the predictions (Figure 1) with the DL method under APC were 0.393, 0.388 and 0.306 for environments 1, 2 and 3, respectively. The corresponding standard errors (SE) for the APCs are given in Table B1 of Appendix B.

### Maize data set 2-trait ASI

**Figure 2:**
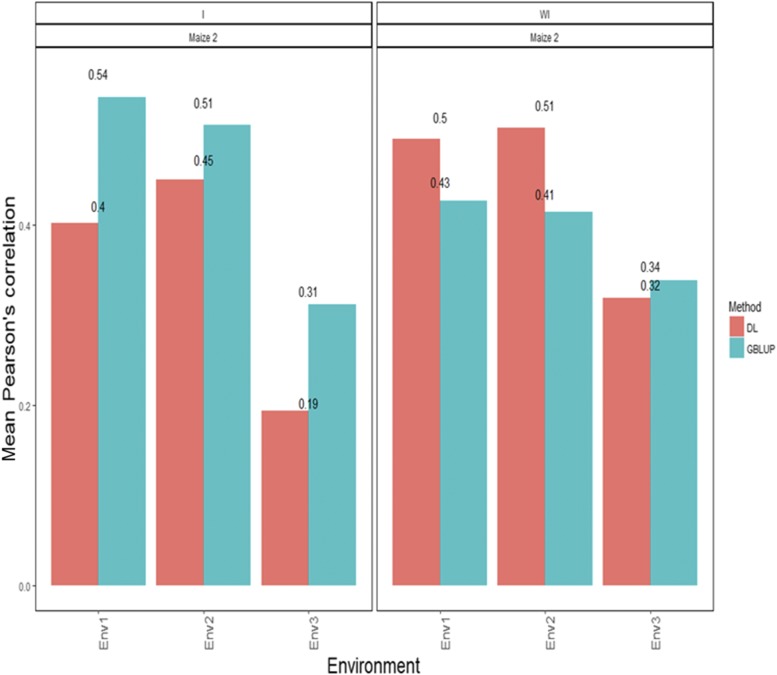
*Maize data set 2- Trait ASI*. Mean Pearson’s correlation for each environment. The first vertical sub-panel corresponds to the model with genotype×environment interaction (Maize data set 2 I), and the second vertical sub-panel corresponds to the same model but without genotype× environment interaction (Maize data set 2 WI).

Figure 2 shows that the APC for each environment for the GBLUP method including the interaction term was 0.542 for environment 1, 0.512 for environment 2 and 0.312 for environment 3. On the other hand, the predictions (Figure 2) with the DL method with interaction in terms of APC were 0.402 for environment 1, 0.451 for environment 2 and 0.194 for environment 3. On the other hand, when the G×E interaction term was ignored, the predictions of the GBLUP method were 0.427 for environment 1, 0.414 for environment 2 and 0.339 for environment 3. Under the DL method, the predictions (Figure 2) in APC terms were 0.496 for environment 1, 0.509 for environment 2 and 0.319 for environment 3 (Figure 2).

### Maize data set 3-trait PH

**Figure 3:**
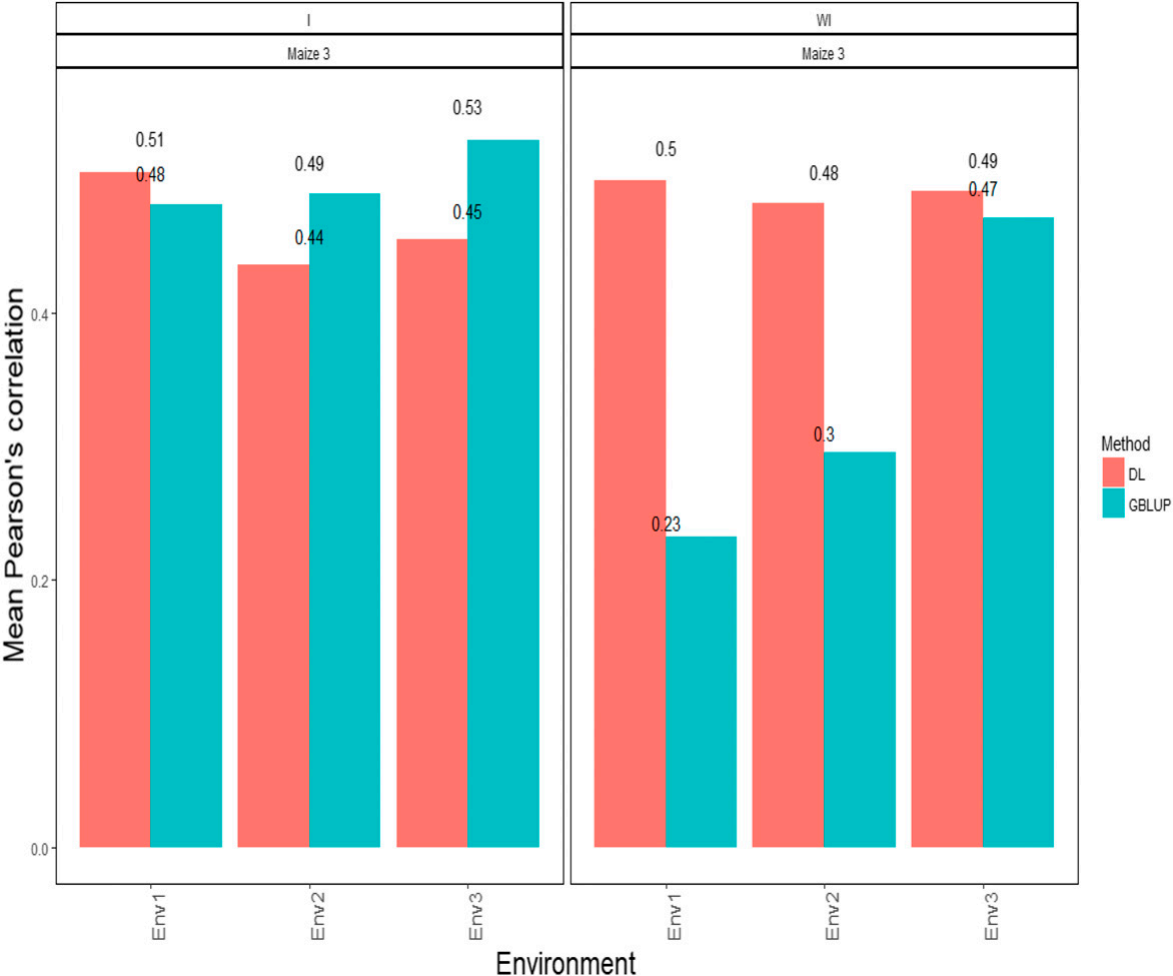
*Maize data set 3- Trait PH*. Mean Pearson’s correlation for each environment. The first vertical sub-panel corresponds to the model with genotype×environment interaction (Maize data set 3 I), and the second vertical sub-panel corresponds to the same model but without genotype× environment interaction (Maize data set 3 WI).

Figure 3 shows that the APCs for the GBLUP method with the G×E interaction term were: 0.481 for environment 1, 0.489 for environment 2 and 0.529 for environment 3. On the other hand, the predictions (Figure 3) obtained with the DL method under the APC were: 0.506 for environment 1, 0.436 for environment 2 and 0.455 for environment 3. When the G×E interaction term was not taken into account, the APCs of the GBLUP method were: 0.232 for environment 1, 0.296 for environment 2 and 0.471 for environment 3. Under the DL method, the predictions (Figure 3) resulting in APC terms were 0.499 for environment 1, 0.482 for environment 2 and 0.491 for environment 3 (Figure 3).

### Wheat data set 4-trait GY

**Figure 4:**
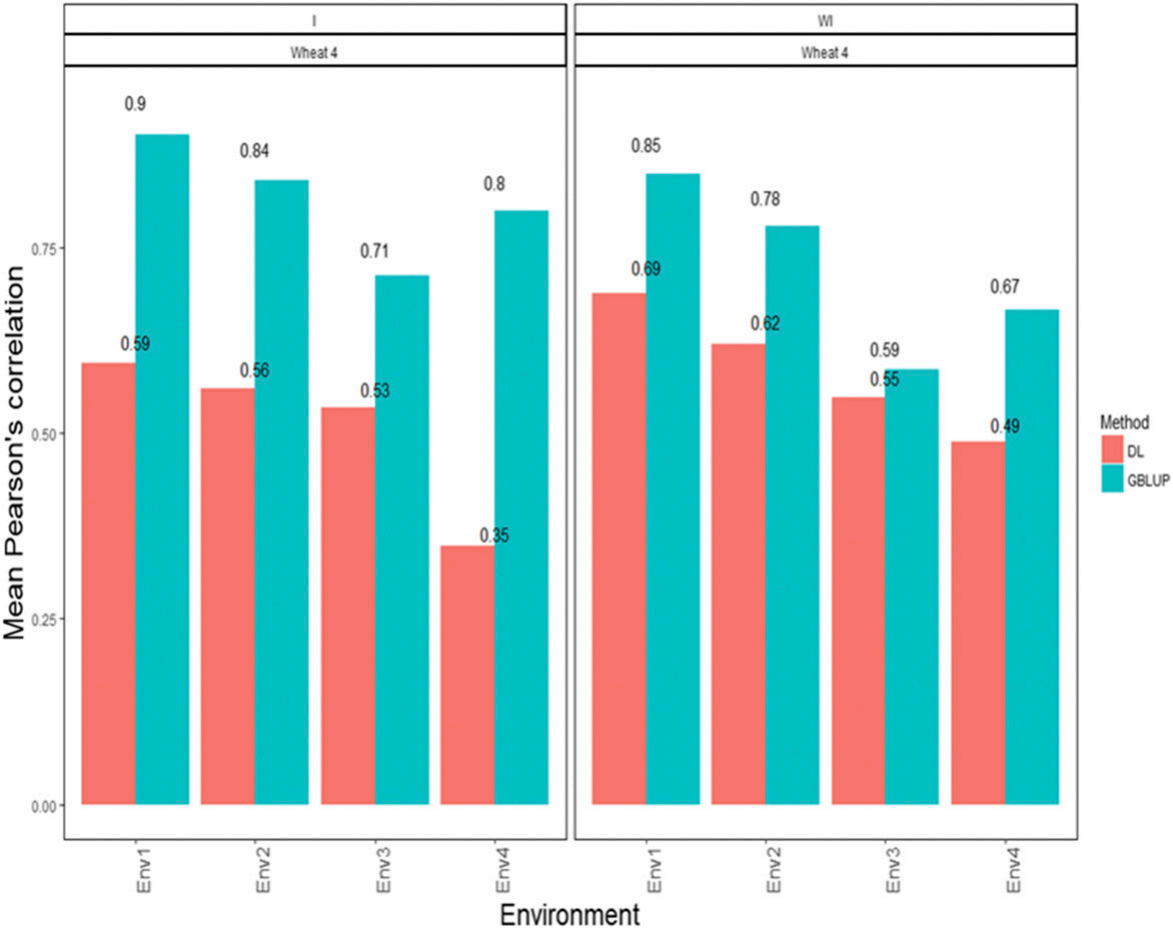
*Wheat data set 4- Trait GY*. Mean Pearson’s correlation for each environment. The first vertical sub-panel corresponds to the model with genotype×environment interaction (Wheat data set 4 I), and the second vertical sub-panel corresponds to the same model but without genotype×environment interaction (Wheat data set 4 WI).

The predictions with the APC for each environment under the GBLUP method with the interaction term were 0.902 for environment 1, 0.841 for environment 2, 0.712 for environment 3 and 0.800 for environment 4 (Figure 4). On the other hand, the predictions (Figure 4) with the APC under the DL method were 0.594 for environment 1, 0.559 for environment 2, 0.534 for environment 3 and 0.348 for environment 4 (Figure 4). When the G×E interaction was ignored, the predictions under the GBLUP method were 0.848 for environment 1, 0.779 for environment 2, 0.585 for environment 3 and 0.666 for environment 4, while under the DL method, the predictions (Figure 4) were 0.689 for environment 1, 0.620 for environment 2, 0.548 for environment 3 and 0.488 for environment 4 (Figure 4).

### Wheat data set 5-trait GY

**Figure 5:**
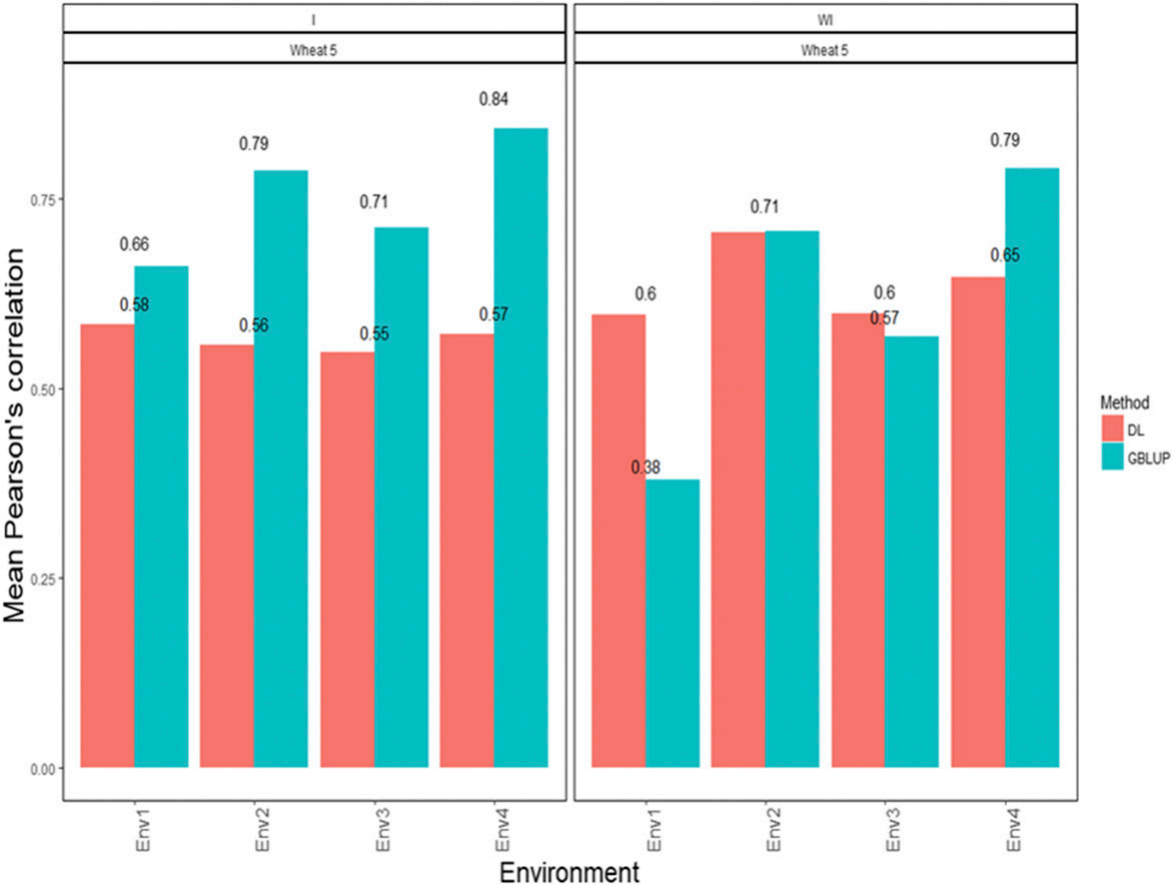
*Wheat data set 5-Trait GY*. Mean Pearson’s correlation for each environment. The first vertical sub-panel corresponds to the model with genotype×environment interaction (Wheat data set 5 I), and the second vertical sub-panel corresponds to the same model but without genotype×environment interaction (Wheat data set 5 WI).

Figure 5 shows that the APCs under the GBLUP method disaggregated by environment with the G×E interaction term were 0.661 for environment 1, 0.787 for environment 2, 0.713 for environment 3 and 0.843 for environment 4 (Figure 5). On the other hand, the predictions (Figure 5) under the DL method were 0.584 for environment 1, 0.557 for environment 2, 0.548 for environment 3 and 0.571 for environment 4 (Figure 5). When the G×E term was ignored, the predictions for the GBLUP method were 0.380 for environment 1, 0.707 for environment 2, 0.568 for environment 3 and 0.791 for environment 4, while the predictions (Figure 5) under the DL method were 0.597 for environment 1, 0.706 for environment 2, 0.599 for environment 3 and 0.647 for environment 4 (Figure 5).

### Wheat data set 6-trait GY

**Figure 6:**
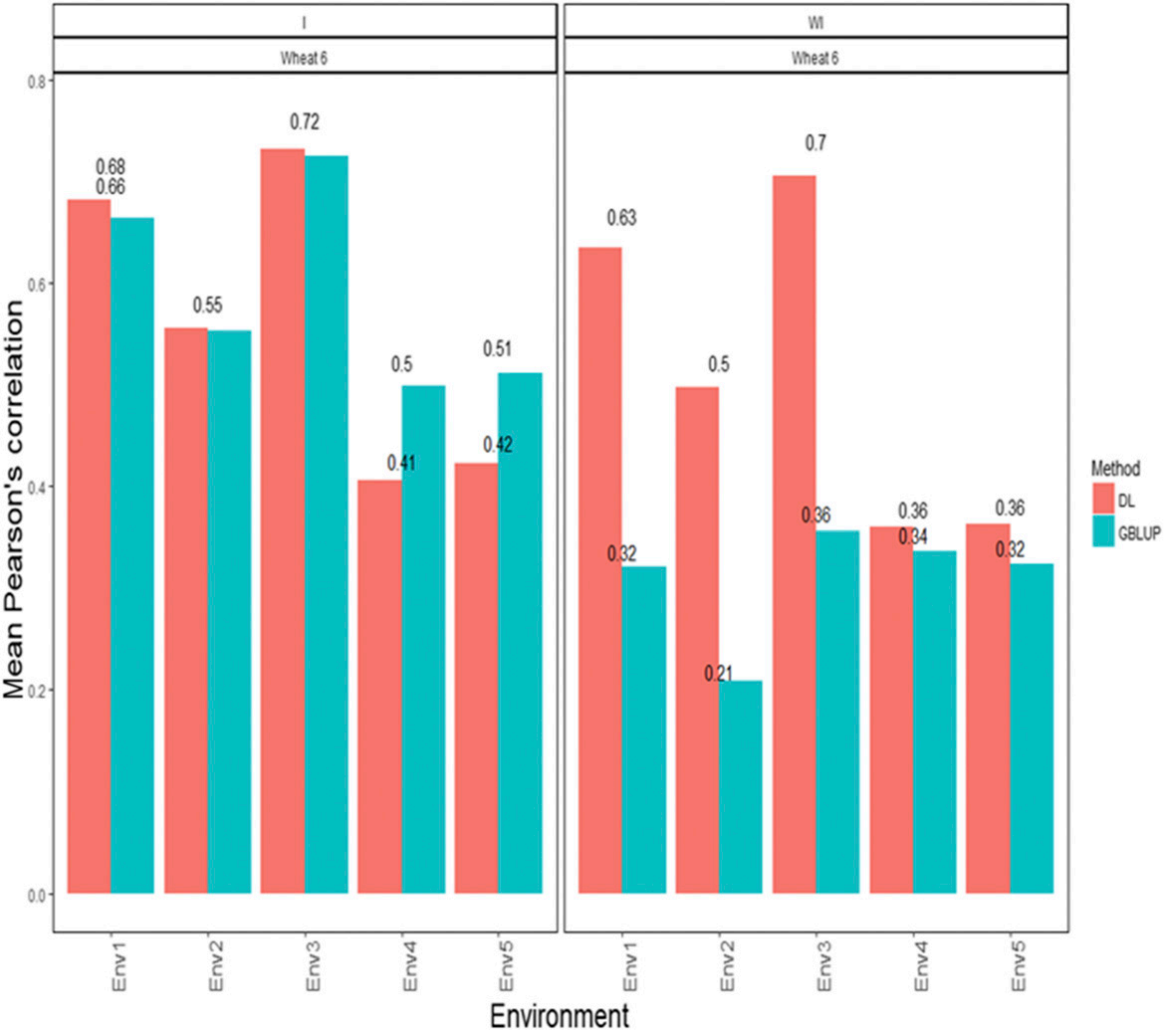
*Wheat data set 6- Trait GY*. Mean Pearson’s correlation for each environment. The first vertical sub-panel corresponds to the model with genotype×environment interaction (Wheat data set 6 I), and the second vertical sub-panel corresponds to the same model but without genotype×environment interaction (Wheat data set 6 WI).

Figure 6 shows that the APCs under the GBLUP method disaggregated by environment with interaction were 0.664 for environment 1, 0.552 for environment 2, 0.724 for environment 3, 0.498 for environment 4 and 0.511 for environment 5 (Figure 6). Under the DL method with the G×E interaction term, the predictions (Figure 6) in terms of APC were 0.682 for environment 1, 0.555 for environment 2, 0.731 for environment 3, 0.405 for environment 4 and 0.422 for environment 5 (Figure 6). When the G×E term was ignored under the GBLUP, the predictions were 0.321 for environment 1, 0.209 for environment 2, 0.356 for environment 3, 0.337 for environment 4 and 0.324 for environment 5 (Figure 6). Under the DL method, the predictions in terms of APC were 0.634 for environment 1, 0.497 for environment 2, 0.705 for environment 3, 0.359 for environment 4 and 0.363 for environment 5 (Figure 6).

### Wheat data set 7-trait PH

**Figure 7:**
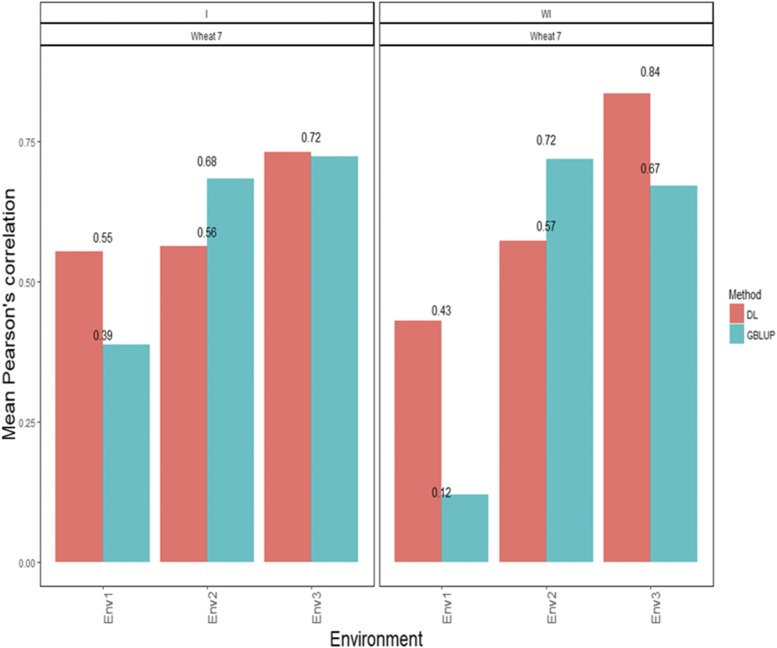
*Wheat data set 7- Trait PH*. Mean Pearson’s correlation for each environment. The first vertical sub-panel corresponds to the model with genotype×environment interaction (Wheat data set 7 I), and the second vertical sub-panel corresponds to the same model but without genotype×environment interaction (Wheat data set 7 WI).

Figure 7 shows that the APCs for the GBLUP method were 0.388 for environment 1, 0.684 for environment 2, and 0.724 for environment 3 (Figure 7). The predictions (Figure 7) under the DL method with interaction in terms of Pearson’s correlation were 0.554 for environment 1, 0.563 for environment 2 and 0.733 for environment 3 (Figure 7). On the other hand when the interaction term was ignored, the APCs under the GBLUP method were 0.119 for environment 1, 0.719 for environment 2 and 0.672 for environment 3 (Figure 7). Under the DL method, the predictions in terms of APC were 0.430 for environment 1, 0.573 for environment 2 and 0.836 for environment 3 (Figure 7).

### Wheat data set 8-trait DTHD

**Figure 8:**
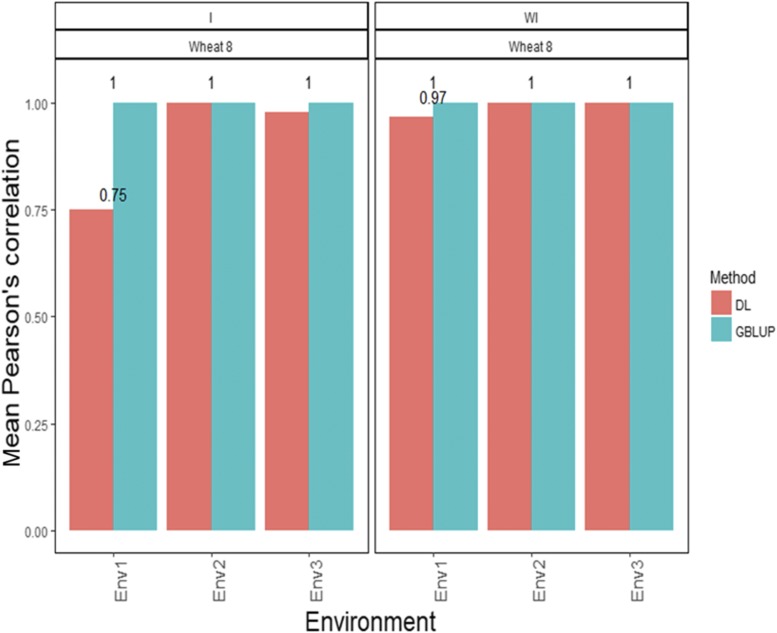
*Wheat data set 8- Trait DTHD*. Mean Pearson’s correlation for each environment. The first vertical sub-panel corresponds to the model with genotype×environment interaction (Wheat data set 8 I), and the second vertical sub-panel corresponds to the same model but without genotype×environment interaction (Wheat data set 8 WI).

Figure 8 shows that the APCs of the GBLUP method with G×E interaction were 1.00 for environment 1, 1.00 for environment 2 and 1.00 for environment 3 (Figure 8). Under the DL method with G×E interaction, the predictions (Figure 8) in terms of APC were 0.75 for environment 1, 1.00 for environment 2 and 0.978 for environment 3 (Figure 8). When the G×E interaction term was ignored, the APCs for environments 1, 2 and 3 were 1.00, 1.00 and 1.00, respectively, under the GBLUP method, while the predictions (Figure 8) under the DL method were 0.967 for environment 1, 1.00 for environment 2 and 1.00 for environment 3 (Figure 8).

### Wheat data set 9-trait DTM

**Figure 9:**
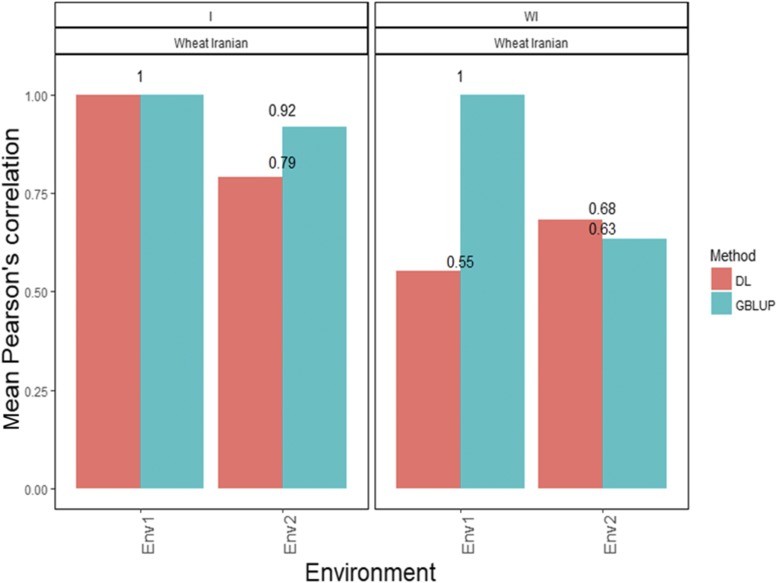
*Wheat data set 9- Trait DTM*. Mean Pearson’s correlation for each environment. The first vertical sub-panel corresponds to the model with genotype×environment interaction (Wheat data set 9 I), and the inferior horizontal sub-panels correspond to the same model but without genotype×environment interaction (Wheat data set 9 WI).

Figure 9 shows that the APCs of the GBLUP method with interaction were 1.00 for environment 1 and 0.918 for environment 2 (Figure 9). Under the DL method, the predictions (Figure 9) in terms of APC were 1.00 for environment 1 and 0.792 for environment 2 (Figure 9). On the other hand, when the interaction term was ignored, the APCs for environments 1 and 2 were 1.00 and 0.633, respectively, under the GBLUP method without interaction (Figure 9). The predictions under the DL without G×E method for DTM were 0.633 for environment 2 and 0.552 for environment 1 (Figure 9).

### A meta-picture of the DL method *vs.* the GBLUP model

**Figure 10:**
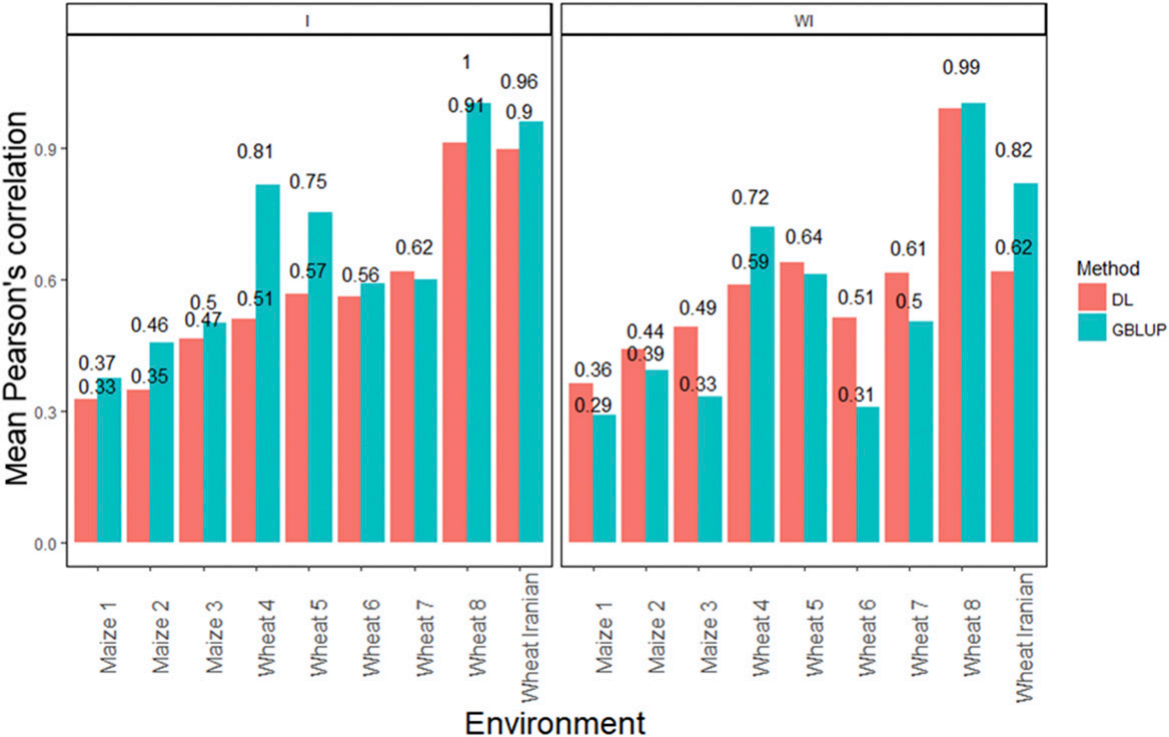
Mean Pearson’s correlation across environments for the GBLUP and the DL model. The first vertical sub-panel corresponds to the model with genotype×environment interaction (I), and the inferior horizontal sub-panels correspond to the same model but without genotype×environment interaction (WI).

Figure 10 shows the mean Pearson’s correlation across environments of the GBLUP model and DL model, with and without G×E interaction for each data set. Here it is evident that for data sets 1, 2, 3, 5, 6 and 7 when the G×E interaction term was not taken into account, the DL method was better than the GBLUP model. When the G×E interaction term was taken into account, the GBLUP model was the best in 8 out of 9 of data sets under study; only in data set 7, the DL method was better than the GBLUP model.

## Discussion

The rapid increase in the genomic data dimension and acquisition rate is challenging conventional genomic analysis strategies. The DL method that recently appeared in the biological arena promises to leverage very large data sets to find hidden structures within them, and make accurate predictions (Angermueller *et al.*, 2016). In other words, DL algorithms dive into data in ways that humans cannot, detecting features that might otherwise be impossible to catch. In our study, we explored a fraction of all possible combinations of hyperparameters of DL methods. Based on our results, we found that the DL method with densely connected network architecture competes well with the GBLUP method, since in many scenarios under study we did not find great differences between these two approaches. The network structure implemented with the DL method is a feedforward multilayer neural network whose structure (topology) is composed of an input layer, one or many hidden layers, and a single output layer. Each layer can have a different number of neurons and each layer is fully connected to the adjacent layer. The connections between the neurons in the layers form an acyclic graph.

One possible explanation for the good performance of the GBLUP method compared with the DL method is that, as has been documented, when the data are scarce (no really large data sets in terms of observations), many times the most commonly used statistical (or machine) learning method outperforms the DL method. Given that with small data sets, one of the major challenges when training a DL method is dealing with the risk of overfitting (*i.e.*, when the training error is low but the testing error is high), the method fails to learn a proper generalization of the knowledge contained in the data. For this reason, in our application of DL with a densely connected network, we used dropout regularization, which consists of temporarily removing a random subset (30%) of neurons with their connections during training. However, even with regularization, DL results were not superior in general terms to GBLUP results when the interaction term was taken into account.

It is important to point out that the DL method was superior when the G×E interaction term was not included in the method under the grid of parameters implemented. This can be attributed to the fact that DL methods are capable of capturing complex relationships hidden in the data without requiring strong assumptions about the underlying mechanisms, which are frequently unknown or insufficiently defined (Angermueller *et al.* 2016). Also, DL methods are a type of general-purpose approach for learning functional relationships from data that do not require prior information, as do the GBLUP and other genomic Bayesian methods. However, three main disadvantages of DL are: (a) it is really hard to train a DL method because we need to test different combinations of hyperparameters corresponding to the number of layers, the number of units, the number of epochs, the type of regularization (and the dropout percentage in the context of dropout regularization) and the type of activation function in each layer; (b) the computational time required to implement a DL method, since it increases as the number of layers and units increases; and (c) a DL method requires a level of experience in computer science and statistics that is not always available in organizations working with biological data.

Also, according to our results, the best combination of hyperparameters (*i.e.*, number of layers, number of units and number of epochs) is data dependent since the best prediction in each data set can be obtained with a different combination of hyperparameters, which corroborates that the process of hyperparameter tuning in DL is a challenging process that required further investigation.

Based on our results, the DL methods are a powerful complement of classic genomic-enabled prediction tools and other analysis strategies. For these reasons, DL methods have been applied successfully in many areas of science, from social science to engineering. However, the results obtained here only apply to DL methods with densely connected network architecture and for the studied hyperparameters; but there are still opportunities to evaluate the performance of other network architectures such as convolutional neural networks and recurrent neural networks.

Furthermore, in the companion article of Montesinos-López *et al.* (2018) the authors extended the multi-environment DL model of this research to the case of multi-trait multi-environment DL model (MTDL) and found challenging aspects for the selection of the hyperparameters. However, the authors have concluded that that MTDL is feasible, and practical in the GS framework with important savings on computing resources as compared to other multi-trait multi-environment models.

Finally, it should be noted that although the DL method performed well compared to the most popular Bayesian genomic selection method (GBLUP), its prediction accuracy was always lower than that of the GBLUP method. However, the boom of DL methods is very widespread and the media are selling DL as the panacea for predicting any type of phenomenon. However, as pointed out above, the DL method also has many limitations that need to be improved, since it is a methodology with a rational thought process that is entirely dependent on the problem we are trying to solve. A lot of time is needed to understand its essence and be able to take advantage of its virtues when trying to apply it to solve real-world problems. However, we must also point out that DL is an alternative approach that can help explore other pathways that underlie biological data.

## Conclusions

In this paper we compare a DL method with densely connected network architecture to the most popular genomic prediction method, the GBLUP. Our results show that the DL method with densely connected network architecture performed as well as the GBLUP method, but that in general terms, the GBLUP method was superior when the covariates corresponding to G×E interaction were taken into account. However, the DL method was superior (in terms of Pearson’s correlation) to the GBLUP method when G×E interaction was ignored, since in 6 out of the 9 data sets under this scenario, the DL method was better than the GBLUP method in terms of prediction accuracy. Based on this empirical evidence, we can say that DL methods with densely connected network architecture were competitive with the most popular genomic prediction method (GBLUP). For this reason, DL methods should be added to the data science toolkit of statisticians, animal and plant breeding scientists so they can use them to evaluate other data sets and other types of network architectures of DL methods that have been applied successfully in other scientific domains.

## Appendix

### Deep learning R codes for a densely connected network

setwd(“C:\\TELEMATICA 2017\\Deep Learning CONTINUOUS”)

rm(list = ls())

######Libraries required##################################

library(tensorflow)

library(keras)

#############Loading data###############################

load(“Data_Maize_1to3.RData”)

####Genomic relationship matrix (GRM)) and phenotipic data#####

G=G_maize_1to3

Pheno=Pheno_maize_1to3

head(Pheno)

###########Cholesky decomposition of the GRM##############

LG=t(chol(G))

########Creating the desing matrices ########################

Z1G=model.matrix(∼0+as.factor(Pheno$Line))

ZE=model.matrix(∼0+as.factor(Pheno$Env))

Z1G=Z1G%*%LG ####Incorporating marker information to lines

Z2GE=model.matrix(∼0+as.factor(Pheno$Line):as.factor(Pheno$Env))

G2=kronecker(diag(3),data.matrix(G))

LG2=t(chol(G2))

Z2GE=Z2GE%*%LG2

###Defining the number of epoch and units#####################

units_M=50

epochs_M=20

##########Data for trait GY#################################

y =Pheno$Yield

X = cbind(ZE, Z1G, Z2GE)

#############Training and testing sets########################

n=dim(X)[1]

Post_trn=sample(1:n,round(n*0.65))

X_tr = X[Post_trn,]

X_ts = X[-Post_trn,]

y_tr = scale(y[Post_trn])

Mean_trn=mean(y[Post_trn])

SD_trn=sd(y[Post_trn])

y_ts = (y[-Post_trn]- Mean_trn)/SD_trn

#########Model fitting in Keras################################

model <- keras_model_sequential()

#########Layers specification ################################

model %>%

layer_dense(

units =units_M,

activation = “relu”,

input_shape = c(dim(X_tr)[2])) %>%

layer_dropout(rate = 0.3) %>% ###Input Layer

layer_dense(units = units_M, activation = “relu”) %>%

layer_dropout(rate = 0.3) %>% ###Hidden layer 1

layer_dense(units = units_M, activation = “relu”) %>%

layer_dropout(rate = 0.3) %>% ####Hidden layer 2

layer_dense(units = 1) ####Output layer

model %>% compile(

loss = “mean_squared_error”,

optimizer = optimizer_adam(),

metrics = c(“mean_squared_error”))

history <- model %>% fit(

X_tr, y_tr, epochs = epochs_M, batch_size = 30,

verbose = FALSE)

#######Evaluating the performance of the model###################

pf = model %>% evaluate(x = X_ts, y = y_ts, verbose = 0)

y_p = model %>% predict(X_ts)

y_p=y_p*SD_trn+ Mean_trn

y_ts=y_ts

y_ts=y_ts*SD_trn+ Mean_trn

###############Observed and predicted values of the testing set#

Y_all_tst = data.frame(cbind(y_ts, y_p))

cor(Y_all_tst[,1],Y_all_tst[,2])

plot(Y_all_tst)

## APPENDIX B

**Table B1.** Standard errors (SE) for the average Pearson’s correlation (APC) for each environment in each of the 9 data sets. I denotes with and WI denotes without the (G×E) interaction term

Env	Method	Interaction	Maize 1	Maize 2	Maize 3	Wheat 4	Wheat 5	Wheat 6	Wheat 7	Wheat 8	Wheat 9
Env1	GBLUP	I	0.040	0.030	0.058	0.008	0.015	0.014	0.021	0.011	0.008
Env2	GBLUP	I	0.042	0.036	0.031	0.015	0.024	0.019	0.019	0.012	0.008
Env3	GBLUP	I	0.044	0.032	0.023	0.019	0.015	0.007	0.022	0.016	-
Env4	GBLUP	I	-	-	-	0.011	0.015	0.014	-	-	-
Env5	GBLUP	I	-	-	-	-	-	0.013	-	-	-
Env1	DL	I	0.036	0.036	0.037	0.016	0.017	0.017	0.034	0.030	0.005
Env2	DL	I	0.040	0.028	0.033	0.013	0.033	0.013	0.021	0.010	0.016
Env3	DL	I	0.083	0.066	0.028	0.021	0.027	0.013	0.025	0.016	-
Env4	DL	I	-	-	-	0.036	0.024	0.030	-	-	-
Env5	DL	I	-	-	-	-	-	0.031	-	-	-
Env1	GBLUP	WI	0.048	0.038	0.073	0.010	0.010	0.035	0.049	0.010	0.007
Env2	GBLUP	WI	0.079	0.042	0.066	0.017	0.026	0.050	0.013	0.011	0.015
Env3	GBLUP	WI	0.044	0.036	0.028	0.014	0.024	0.032	0.020	0.016	-
Env4	GBLUP	WI	-	-	-	0.016	0.020	0.022	-	-	-
Env5	GBLUP	WI	-	-	-	-	-	0.033	-	-	-
Env1	DL	WI	0.029	0.028	0.040	0.018	0.018	0.021	0.052	0.020	0.015
Env2	DL	WI	0.039	0.032	0.039	0.019	0.024	0.028	0.030	0.008	0.017
Env3	DL	WI	0.063	0.041	0.036	0.028	0.018	0.010	0.025	0.009	-
Env4	DL	WI	-	-	-	0.019	0.020	0.038	-	-	-
Env5	DL	WI	-	-	-	-	-	0.030	-	-	-
